# 

**Published:** 1898-06

**Authors:** 


### CONTENTS.

ORIGINAL ARTICLES: PAGE
Sanitation versus Tuberculin in the Eradication of Tuberculosis. By G. N. KINNELL, M.R.C.V.S. . . . 369
Toe-divided Shoes for Contracted Hoofs. By M. J. TREACY, M.R.C.V.S. . . . 377
ABSTRACTS FROM FOREIGN JOURNALS:
Roentgen Rays as a Remedy for Articular Rheumatism . . . 379
The Question of Raising the Official Rank of District Veterinarians in Austria . . 379
Spontaneous Recovery from a Chronic Prolapsus Penis in the Horse . . . 380
A New Tattooing Apparatus . . . 381
The Growth of Animals . . . 381
Researches as to the Value of Mallein as a Diagnostic Agent in Glanders . . . 382
SELECTIONS:
For Pure Food . . . 383
Wolf-in-the-Tail . . . 385
REPORTS OF CASES:
Abscess of the Sheath of the Flexor Pedis Perforans Tendon. By W. J. MARTIN, V.S. 389
Reports of Odd Cases. By E. L. QUITMAN, M.D.C. . . . 391
Clinical Notes from the McKillip Veterinary College, Chicago, Ill. . . . 395
Clinical Notes. By HUGH THOMPSON, V.S. . . . 396
Purpura Hemorrhagica treated with Antistreptococcus Serum; Recovery. By L. R. WEBBER . . . 398
Fracture of Penis: Amputation; Recovery. By N. B. SMITH, D.V.M. . . . 399
AMONG THE PROFESSION IN ILLINOIS . . . 400
The Prairie State Veterinary Colleges . . . 402
Illinois Associations—About Illinois State Laws . . . 404
What Illinois Veterinarians Need . . . 405
A Card of a "Windy City" Specialist . . . 406
EDITORIALS:
What will the Veterinarians of Illinois Do?—Illinois Opportunity . . . 407
Points in Regard to Army Legislation . . . 408
Another Aspirant for Veterinary Fame—Fools Rush in Where Angels Fear to Tread —Dr. Salmon's Report on Government Meat-inspection—Report of the National Stock-grower's Convention . . . 409
Another Curiosity—Marriages—Necrology—Correction . . . 410
CONTROL WORK:
Report of the Veterinary Department of the Minnesota State Board of Health for Quarter and Year Ending, January 1, 1898 (concluded)—Illinois . . . 411
SOCIETY PROCEEDINGS . . . 422
COMMENCEMENT EXERCISES . . . 435
PERSONALS—POINTS . . . 437

